# Hepatic rupture: A case report of a severe complication of percutaneous catheter drainage

**DOI:** 10.1097/MD.0000000000009499

**Published:** 2018-01-12

**Authors:** Liang Zhang, DaLong Wan, LeLe Zhang, ShiGuo Xu, HaiYang Xie, ShengZhang Lin

**Affiliations:** a Department of Hepatobiliary and Pancreatic Surgery, the First Affiliated Hospital, Zhejiang University School of Medicine, Hangzhou; b Key Lab of Combined Multi-Organ Transplantation, Ministry of Public Health, Key Lab of Organ Transplantation, P.R. China.

**Keywords:** case report, complication, hepatic rupture, management, percutaneous catheter drainage

## Abstract

**Rationale:**

Currently, percutaneous catheter drainage (PCD) is regarded as the first-line treatment modality of pyogenic liver abscess. Severe complications associated with PCD were uncommon. Hepatic rupture is an uncommon but life-threatening liver trauma with high mortality. Its management is challenging because a delay in the diagnosis may lead to fatal hemorrhagic shock. To our knowledge, PCD-associated hepatic rupture has never been reported.

**Patient concerns:**

We report herein a rare case of PCD-associated hepatic rupture. Its clinical courses and our therapeutic approaches are presented. Moreover, the clinical significance, underlying causes, and current views on severe liver trauma management will be discussed briefly.

**Diagnoses:**

A diabetic patient suffering from fever and malaise was diagnosed with a pyogenic liver abscess. PCD was performed because intravenous antibiotics were ineffective. The patient developed a liver rupture following PCD, with clinical and imaging confirmation but without further progression.

**Interventions:**

Surgical repair and vascular intervention were both inappropriate. As a result, medical treatments with supportive care were adopted and were found to be effective.

**Outcomes:**

The patient's condition improved gradually, with stabilized imaging and laboratory performance. He recovered uneventfully during follow-ups.

**Lessons:**

Hepatic rupture should be listed as an extremely rare but severe complication of PCD. Immediate suspicion and effective intervention may avoid an unfavorable consequence.

## 1 Introduction

It has been widely accepted that percutaneous catheter drainage (PCD), the first-line treatment modality of pyogenic liver abscess, is characterized by minimal invasiveness, high success rates, and well-established safety.^[1]^ Severe complications associated with PCD are uncommon.^[2,3]^ Furthermore, hepatic rupture is an uncommon but life-threatening liver injury with high mortality. Its management remains challenging because a delay in diagnosis may lead to fatal hemorrhagic shock. Liver rupture has not been described in association with PCD in the literature to date. Recently, we encountered an extremely rare case of liver rupture following PCD and managed it successfully. Herein, we present the clinical course of the case and our management approaches. The clinical significance, underlying causes, and current views on severe liver trauma management will also be discussed briefly.

## 2 Case report

**Figure 1:**
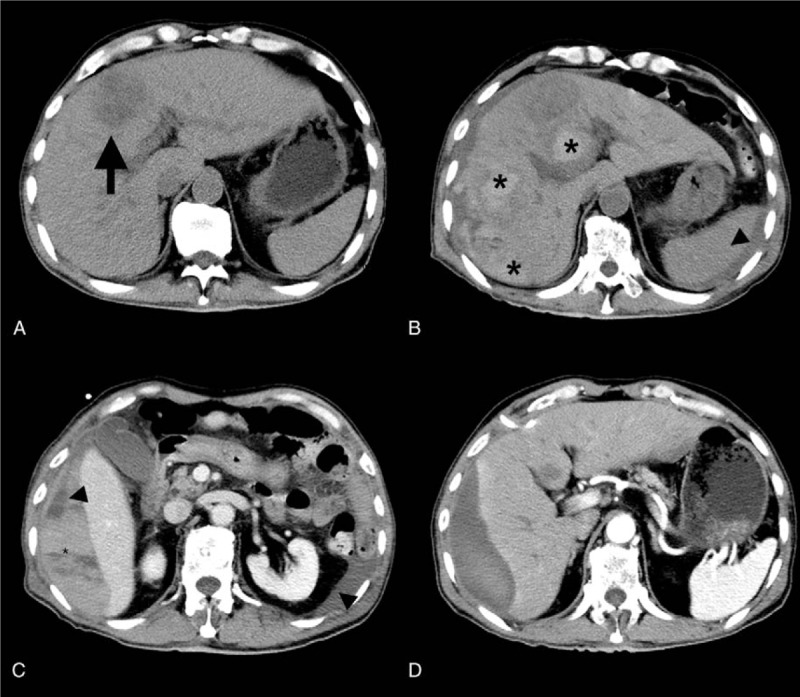
CT images before and after PCD. A. CT images on 3 days before PCD: a 4 cm liver abscess located in segment IV (arrow). B, C. CT images on 2 days after PCD: subcapsular and intrahepatic hematoma (asterisk), subcapsular and intraabdominal hypo-intense fluid collection (arrowhead), residual liver abscess and drainage catheter shedding. D. CT images on 34 days after PCD: repeated CT revealed hematoma and ascites had been absorbed significantly, abscess cavity closure with residual subcapsular fluid accumulation. CT = computed tomography, PCD = percutaneous catheter drainage.

A 71-year-old man was admitted with a complaint of episodic fever for 1 week, accompanied by general malaise, nausea, and vomiting. His past medical history was negative, except for uncontrolled diabetes mellitus. On initial admission, his body temperature was 39.5 °C. Physical examination was unremarkable. Laboratory tests showed white blood cell counts 16 × 10^9^/L, neutrophil 91.6%, hemoglobin 116 g/L, C-reactive protein 134.4 mg/L, fasting blood glucose 17 mmol/L, and normal liver function tests (LFTs). Blood culture, tumor, and viral markers were all negative. Abdominal computed tomography (CT) scans revealed a 5 cm abscess located in liver segment IV (Fig. 1A). Insulin was administered subcutaneously to control his hyperglycemia. The initial broad-spectrum antibiotic therapy we had employed (cefoperazone sodium and ornidazole, intravenously) was ineffective; therefore, ultrasound guided PCD was attempted. After topical anesthesia with 2% solution of lidocaine hydrochloride, an 8-French pigtail catheter was introduced into the abscess cavity using the Seldinger technique, under ultrasound guidance. Pus was successfully drained; *Burkholderia vietnamiensis* was found in the pus culture. Within 24 hours following PCD, the patient complained of severe right upper quadrant pain. Three hundred milliliter hematic fluid was drained from the catheter. His hemoglobin and hematocrit levels continuously decreased (from 104 to 65 g/L and from 29.1% to 18.7%, respectively). LFTs revealed elevated liver enzymes: alanine aminotransferase 654U/L and aspartate aminotransferase 1210 U/L; while his hemodynamic status remained stable: blood pressure 112/83 mmHg, pulses 75 beats/min, Spo2 96% to 100% (room air). Creatinine, bilirubin, urinalysis, coagulation function tests, and plate counts were all within normal limits. Ultrasound indicated a 10 × 4 cm subcapsular hematoma in the right lobes. Abdominal enhanced CT further revealed intrahepatic and subcapsular hematoma extending from the right diaphragmatic face to the hilum, intra-abdominal fluid accumulation, and catheter shedding (Fig. 1B and C). Subsequently, emergent angiogram was performed, and revealed an absence of intrahepatic artery-venous fistula or active bleeding signs. According to these findings, a diagnosis of liver rupture without further progression was made. Under close monitoring, the patient accepted transfusions of 2 units of packed red blood cells, with hemostatic drug usage (aminomethylbenzoic acid and etamsylate, intravenously). The patient's antibiotic regimen was adjusted (imipenem and cilastatin, intravenously) according to pus culture sensitivity. After these conservative treatments continued for 5 days, the patient's fever subsided, and discomfort resolved gradually. Laboratory workup revealed that his hemoglobin had stabilized and LFTs normalized. The drainage fluid became yellow and clear. Thirty-four days after PCD, repeated abdominal enhanced CT disclosed that the previous hematoma had been absorbed significantly, with ascites resolution and abscess cavity closure (Fig. 1D). The local daily drainage showed a decreased volume, and the catheter was removed. The patient recovered uneventfully during following-ups.

## 3 Discussion

PCD is currently regarded as the first-line treatment modality for pyogenic liver abscess. In a recently published retrospective study, the success rate of PCD for pyogenic liver abscesses reached 95%.^[4]^ A meta-analysis, enrolling 5 randomized controlled trials, has demonstrated that PCD is more effective than percutaneous needle aspiration.^[5]^ Despite the established safety of this procedure, several cases involving serious PCD-related complications have been reported, including hepatocolic fistula and hemobilia.^[2,3]^ Hepatic rupture is frequently associated with blunt liver trauma. Other uncommon causes include obstetric diseases, coagulopathy and, rarely, peliosis hepatis.^[6–8]^ To our knowledge, PCD-associated hepatic rupture has not been reported before.

Severe liver trauma is a life-threatening situation with a high mortality rate of 4.0% to 11.7%,^[9]^ and its management is challenging due to emergency. Hepatic rupture, although uncommon, should be regarded as an even more urgent hepatic trauma because a delay in diagnosis may lead to fatal hemorrhagic shock. However, there are no established grading or classification systems currently available for hepatic rupture. Extrapolating from the American Association for the Surgery of Trauma (AAST) grading scale for liver trauma,^[10]^ our case would at least be classified as an AAST grade III liver injury. Recently, some authors have suggested that surgery is not necessary for liver trauma patients without hemodynamic instability.^[11,12]^ Even so, little is known about whether this treatment strategy is still applicable for iatrogenic liver rupture. Previously, there had been few reports of hepatic rupture managed by surgical and interventional approaches.^[13]^ In this case, the operation was not scheduled, mainly due to the low success rate of surgical repair for such extensive intrahepatic hemorrhage. Hepatic arterial embolization was also excluded because there was no contrast medium extravasation sign on angiography. Regarding both hemodynamic stability and serial examination findings, which indicated that the hematoma was not developing further, we initially managed our patient with medical treatments, which also turned out to be successful.

Unfortunately, the triggers that led PCD to cause such an extensive intrahepatic hemorrhage remained relatively obscure and untraceable in our presented case. Diabetes mellitus and abscess formation were both important preconditions that made hepatic tissues brittle. Slipping of the PCD catheter in the poorly liquefied abscess further worsened the situation. Nonetheless, a more optimized explanation with compelling evidence is still needed.

## 4 Conclusions

Hepatic rupture should be listed as an extremely rare but severe complication of PCD, even though PCD is currently regarded as a safe procedure. Conservative treatments under close observation may be effective for those patients not eligible for surgery or vascular intervention but who are in stable circulation states.
